# Multicenter study of carbon‐ion radiation therapy for nonsquamous cell carcinomas of the oral cavity

**DOI:** 10.1002/cam4.2408

**Published:** 2019-08-01

**Authors:** Hiroaki Ikawa, Masashi Koto, Yusuke Demizu, Jun‐ichi Saitoh, Hiroaki Suefuji, Tomoaki Okimoto, Tatsuya Ohno, Yoshiyuki Shioyama, Ryo Takagi, Kazuhiko Hayashi, Kenji Nemoto, Takashi Nakano, Tadashi Kamada

**Affiliations:** ^1^ Hospital of the National Institute of Radiological Sciences National Institutes for Quantum and Radiological Sciences and Technology Chiba Japan; ^2^ Department of Radiology Hyogo Ion Beam Medical Center Tatsuno Japan; ^3^ Gunma University Heavy Ion Medical Center Maebashi Japan; ^4^ Ion Beam Therapy Center SAGA‐HIMAT Foundation Tosu Japan; ^5^ Department of Oral Pathobiological Science and Surgery Tokyo Dental College Tokyo Japan; ^6^ Department of Radiation Oncology, Faculty of Medicine Yamagata University Yamagata Japan

**Keywords:** adenoid cystic carcinoma, carbon‐ion radiotherapy, head and neck cancer, oral cancer, osteoradionecrosis, particle therapy

## Abstract

**Background:**

The aim of this study was to evaluate the efficacy and safety of carbon‐ion radiation therapy for nonsquamous cell carcinomas of the oral cavity in a multicenter study.

**Methods:**

Retrospective analysis of the clinicopathological features and outcomes of 76 patients with oral nonsquamous cell carcinomas with N0‐1 M0 status and were treated with carbon‐ion radiation therapy at four institutions in Japan between November 2003 and December 2014 was performed.

**Results:**

Salivary gland carcinoma, mucosal melanoma, and three other carcinomas were found in 46, 27, and 3 patients, respectively. T1‐3, T4a, and T4b disease was diagnosed in 27, 18, and 31 patients, respectively. Median follow‐up period was 31.1 months (range, 3‐118 months). Three‐year local control, progression‐free survival, and overall survival of all patients were 86.8%, 63.1%, and 78.4%, respectively. Multivariate analysis showed T classification (T4) to be a significant independent poor prognostic factor for local control. Acute grade 3 mucositis was observed in 38 patients. Grades 3 and 4 late morbidities were observed in 9 and 4 patients, respectively. No grade 5 late toxicity was observed.

**Conclusions:**

Oral nonsquamous cell carcinomas could be treated effectively, with acceptable toxicity, by carbon‐ion radiation therapy.

## Graphical abstract

This retrospective multicenter study aimed to evaluate the safety and efficacy of carbon‐ion radiotherapy as a primary treatment for oral malignancies, and our results indicate that most patients had salivary gland carcinoma or mucosal melanoma. The 3‐year rates were 86.4% for local control, 63.1% for progression‐free survival, and 78.4% for overall survival. Oral nonsquamous cell carcinomas could be treated effectively, with acceptable toxicity, by carbon‐ion radiation therapy.

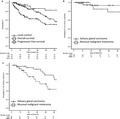

## 1 INTRODUCTION

Oral malignancies, although of various histological types, are mostly squamous cell carcinomas. Furthermore, 6% to 10% of oral malignancies are diagnosed as nonsquamous cell carcinomas.^1^ The standard treatment for oral nonsquamous cell carcinomas is surgery.^2^, ^3^ For inoperable cases, photon radiation therapy (RT) is usually applied. However, nonsquamous cell carcinomas are known to be relatively radio‐resistant, and therefore the role of photon RT for inoperable oral nonsquamous cell carcinomas is limited.^4^, ^5^

Carbon‐ions (C‐ions) provide higher linear energy transfer (LET) and large relative biological effectiveness (RBE) compared with photons, and thus offer a greater possibility of tumor control.^6^ Four carbon‐ion facilities were functioning in Japan by the end of 2014 (Hospital of the National Institute of Radiological Sciences, Hyogo Ion Beam Medical Center, Gunma University Heavy Ion Medical Center, SAGA HIMAT Foundation). A retrospective multicenter study to evaluate the clinical outcomes of C‐ion RT for head and neck malignancies was performed (Japan Carbon‐Ion Radiation Oncology Study Group [J‐CROS] Study: 1402 HN). The clinical outcomes of the respective major histological types of head and neck malignancies have already been reported.^7^, ^8^, ^9^, ^10^, ^11^ The 5‐year local control and overall survival (OS) rates among patients with adenoid cystic carcinoma, mucosal melanoma, and adenocarcinoma of 68% and 74%, 72.3% and 44.6%, and 79.3% and 60.4%, respectively, were also reported.^8^, ^9^, ^11^

Oral malignancies are in close proximity to risk organs, such as oral mucosa and jawbone, compared with other head and neck malignancies. To clarify the efficacy and safety of C‐ion RT for oral nonsquamous cell carcinomas, subanalysis based on primary sites could prove to be clinically useful. Herein, we report the results for a subgroup of patients with oral nonsquamous cell carcinomas using the data of J‐CROS 1402 HN.

## 2 PATIENTS AND METHODS

J‐CROS 1402 HN was designed as a retrospective, multicenter study conducted in four carbon‐ion facilities in Japan (Hospital of the National Institute of Radiological Sciences, Hyogo Ion Beam Medical Center, Gunma University Heavy Ion Medical Center, SAGA‐HIMAT Foundation). It was approved by the institutional review boards of the respective institutes and was conducted in accordance with the Declaration of Helsinki. This trial is registered with UMIN‐CTR (http://www.umin.ac.jp/ctr/index-j.htm), identification number UMIN000024473.

Patients suffering from head and neck malignancies and treated with C‐ion RT between November 2003 and December 2014 were included. Detailed inclusion criteria have been described previously.^7^, ^8^, ^9^, ^10^, ^11^ Patients with previous irradiation to the head and neck were excluded.

The survey included 908 eligible patients. Among them, 76 patients with oral nonsquamous cell carcinomas were enrolled in the study. All tumors were classified by the TNM staging system, seventh edition (International Union Against Cancer, 2009).

### 2.1 Evaluation

No evidence of tumor regrowth in the planning target volume (PTV) was defined as local control, and regional control as no evidence of regional lymph node recurrence or head and neck lesions outside the PTV.

Acute and late toxicities in normal tissues were classified in accordance with the National Cancer Institute Common Terminology of Criteria for Adverse Effect (CTCAE), versions 4.0.

We defined resectability and operability in this study as follows: Resectable and unresectable are determined on a purely technical basis and will depend on the skill and experience of the surgeon. Operable and inoperable will, in addition to resectability, require a consideration of the benefits and risks to the patient, taking into account the age and complications, as to whether a major surgical procedure with its attendant morbidity is justified.

### 2.2 Statistical analyses

Survival times were calculated from the starting day of C‐ion RT. The Kaplan‐Meier method was applied to evaluate the cumulative incidences of local control, progression‐free survival (PFS), and OS. As potential risk factors for local control and OS, age, gender, performance status, tumor status, resectability, T classification, N classification, histology, gross tumor volume (GTV), combined chemotherapy, prescribed dose, and fractionation were all evaluated. Subgroups were compared by univariate log‐rank test. Variables with univariate *P*‐values of .1, two‐sided, were included in a multivariate analysis by Cox proportional hazards model. Differences were considered statistically significant with two‐sided *P*‐values of <.05. All analyses were conducted using IBM SPSS software (version 19; IBM Corp., Armonk, NY).

## 3 RESULTS

### 3.1 Cohort characteristics

We retrospectively analyzed 76 patients with oral nonsquamous cell carcinomas treated with C‐ion RT. Patient and tumor characteristics are summarized in Table 1. All patients were classified with M0 disease. Forty of the resectable cases were inoperable because in all cases, it was impossible to avoid the marked decline in oral function and aesthetics associated with resection. The 40 cases consisted of 18 males and 22 females. Regarding T classification, four cases were T1, 13 cases were T3, 10 cases were T4a, and 13 cases were T4b.

**Table 1:** Patient and tumor characteristics

No. of patients	76
Age (y)	
Median	61.5
Range	31‐80
Sex, n (%)	
Male	31 (41)
Female	45 (59)
Performance status, n (%)	
0	53 (70)
1	23 (30)
Tumor status	
Naive	63 (83)
Recurrence	13 (17)
Resectability, n (%)	
Yes	40 (53)
No	36 (47)
Tumor classification, n (%)	
T1	4 (5)
T2	1 (1)
T3	22 (29)
T4a	18 (24)
T4b	31 (41)
Node classification, n (%)	
N0	72 (95)
N1	4 (5)
Histology, n (%)	
Salivary gland carcinomas	
Adenoid cystic carcinoma	36 (47)
Mucoepidermoid carcinoma	6 (8)
Adenocarcinoma	3 (4)
Acinic cell carcinoma	1 (1)
Mucosal melanomas	27 (36)
Others	
Spindle cell carcinoma	2 (3)
Adenosquamous carcinoma	1 (1)
Gross tumor volume (cm^3^)	
Median	26.9
Range	1.06‐167.5

Treatment characteristics of C‐ion RT for all patients are shown in Table 2. C‐ion RT was performed only on the primary site for N0 cases, and there was no prophylactic irradiation. In four cases in which cervical lymph node metastasis was found before C‐ion RT, involved field was used. Schedule selection depended on the respective institutions. A dose of 64.0 Gy (RBE) in 16 fractions, four fractions per week, was most commonly prescribed. Median treatment duration was 29 days (range, 23‐51).

**Table 2:** Treatment characteristics

Carbon‐ion radiation therapy	
Fractionation, (%)	
16 fractions at 4 fractions per week	57 (75)
26‐32 fractions at 5 fractions per week	19 (25)
Dose and fraction, n (%)	
57.6 Gy (RBE)/16 fractions	27 (36)
64.0 Gy (RBE)/16 fractions	29 (38)
60.8 Gy (RBE)/16 fractions	1 (1)
65.0 Gy (RBE)/26 fractions	11 (14)
70.2 Gy (RBE)/26 fractions	3 (4)
70.4 Gy (RBE)/32 fractions	5 (7)
Number of portals	
Median	5
Range	2‐7

Abbreviations: RBE, relative biological effectiveness.

Twenty‐one patients (27.7%) underwent chemotherapy, which included dimethyl traizeno imidazole carboxamide (DTIC) administered to 17 patients with mucosal melanoma. One patient with spindle cell carcinoma underwent chemotherapy with multiple drugs (cyclophosphamide [CPA], pirarubicin [THP], cisplatin [CDDP]), one patient of adenoid cystic carcinoma underwent gimeracil and oteracil potassium (S‐1). The anticancer drugs in two patients with adenoid cystic carcinoma and mucoepidermoid carcinoma were unknown. Of the 21 patients underwent chemotherapy, 11 patients were administered concurrent chemotherapy, 7 patients were administered neoadjuvant chemotherapy, 2 patients had neoadjuvant and concurrent chemotherapy, and 1 patient had neoadjuvant and adjuvant chemotherapy.

### 3.2 Local control and survival

Median follow‐up time was 31.1 months (range, 3‐118 months). All 7 patients (9.2%) with local recurrence developed it within the PTV. The median interval between C‐ion RT and local recurrence was 20 months (range: 7‐47 months). In these patients, histologies were 4 with ACC, 1 with acinic cell carcinoma, 1 with spindle cell carcinoma, and 1 with malignant melanoma. Regarding T classification, 1 case was T3, 2 cases were T4a, and 4 cases were T4b. Regarding the prescribed dose, 3 cases received 64 Gy (RBE) in 16 fractions, 2 cases 57.6 Gy (RBE) in 16 fractions, 1 case 65 Gy (RBE) in 26 fractions, and 1 case 70.4 Gy (RBE) in 26 fractions. Regional recurrence and distant metastasis developed in 13 (17.1%) and 17 patients (22.4%), respectively. Of the 13 regional recurrence cases after C‐ion RT, 12 had cervical lymph node metastasis, but none had been found to have lymph node metastasis prior to C‐ion RT. The median interval between C‐ion RT and regional recurrence was 18 months (range: 1‐41 months). Of these patients, histologies were mucosal melanoma in 8, ACC in 4, and spindle cell carcinoma in 1. As for T classification, 6 cases were T3, 3 cases were T4a, and 4 cases were T4b. Three cases developed regional + distant metastasis. The most common sites of distant metastasis were lung (n = 7) and brain (n = 3). Nineteen patients died from their disease, and 3 from unrelated causes.

**Figure 1:**
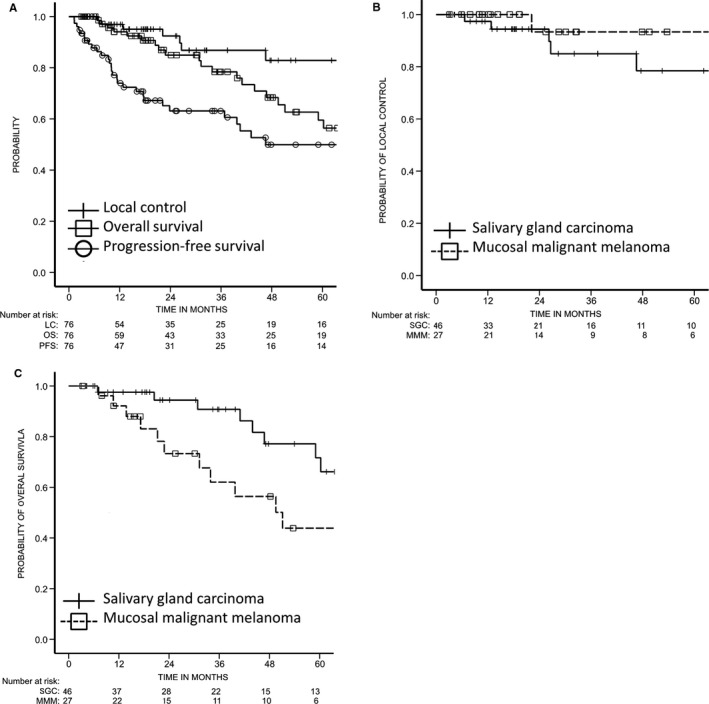
(A) Local control, progression‐free survival (PFS), and overall survival (OS) rates for all patients (n = 76). (B) Local control rates by histological subtypes. The 3‐year local control rates of patients with salivary gland carcinoma and mucosal melanoma were 85.0% and 93.3%, respectively. (C) Overall survival rates by histological subtypes. The 3‐year overall survival rates of patients with salivary gland carcinoma and mucosal melanoma were 90.8% and 62.0%, respectively

Figure 1A shows the cumulative local control, PFS, and OS of all patients. Cumulative 3‐ and 5‐year local control rates were 86.8% (95% confidence interval [CI], 76.6 ‐ 97.0) and 82.9% (95% CI, 70.5 ‐ 95.2), 3‐ and 5‐year PFS rates were 63.1% (95% CI, 51.1 ‐ 75.1) and 49.9% (95% CI, 35.9 ‐ 63.9), and the 3‐ and 5‐year OS rates were 78.4% (95% CI, 67.3 ‐ 89.5) and 59.5% (95% CI, 44.6 ‐ 74.4), respectively.

Figure 1B shows the Kaplan‐Meier survival curves for local control according to histological subtype. The 3‐ and 5‐year local control rates of patients with salivary gland carcinoma (SGC) were 85.0% (95% CI, 70.8 ‐ 99.1) and 78.4% (95% CI, 60.5 ‐ 96.4), respectively. Both 3‐ and 5‐year local control rates of patients with mucosal melanoma were 93.3% (95% CI, 80.7 ‐ 100.0).

The Kaplan‐Meier survival curves for OS by histological subtype are shown in Figure 1C. The 3‐ and 5‐year OS rates of patients with SGC were 90.8% (95% CI, 80.7 ‐ 100.0) and 71.7% (95% CI, 53.1 ‐ 90.3), and those of patients with mucosal melanoma were 62.0% (95% CI, 40.8 ‐ 83.3) and 43.9% (95% CI, 20.9 ‐ 66.8), respectively.

### 3.3 Prognostic factors

Table 3 shows the results of univariate and multivariate analysis of prognostic factors for local control and OS. As for local control, there was no significant difference in resectability of salivary gland carcinoma (*P* = .86) and mucosal melanoma (*P* = .546), respectively, according to univariate analysis. Multivariate analysis showed T‐classification (T4) (hazard ratio [HR] = 10.881, 95% CI = 1.093‐108.363, *P* = .042) to be a significant independent risk factor for local control (Table 3), but it did not reveal any significant risk factors for OS.

**Table 3:** Risk factors for local control and overall survival

Factors	n	Local recurrence			Overall survival		
		Univariate analysis	Multivariate analysis		Univariate analysis	Multivariate analysis	
		*P* value	*P* value	HR (95% CI)	*P* value	*P* value	HR (95% CI)
Age							
<62	38	.444			.081	.086	2.321 (0.888‐6.068)
≥62	38						
Sex							
Male	31	.105			.019	.204	0.469 (0.146‐1.509)
Female	45						
Performance status							
0	53	.342			.460		
1	23						
Tumor status							
Naive	63	.170			.559		
Recurrence	13						
Resectability							
Yes	40	.882			.379		
No	36						
T classification							
T1‐3	27	.077	.042	10.881 (1.093‐108.363)	.830		
T4	49						
N classification							
N0	72	.435			.924		
N1	4						
Histology							
Salivary gland carcinoma	46	<.001	.132	2.893 (0.727‐11.515)	.002	.499	1.477 (0.477‐4.576)
Mucosal melanoma	27						
Others	3						
Gross tumor volume							
<27 ml	39	.497			.719		
≥27 ml	37						
Combined chemotherapy							
No	55	.644			.592		
Yes	21						
Dose							
57.6 Gy (RBE)	27	.709			.363		
64.0 Gy (RBE)	29						
60.8 Gy (RBE)	1						
65.0 Gy (RBE)	11						
70.2 Gy (RBE)	3						
70.4 Gy (RBE)	5						
Fractionation							
16 fractions	57	.531			.689		
>16 fractions	19						

Abbreviations: CI, confidence interval; HR, hazard ratio; N, node; RBE, relative biological effectiveness; T, tumor.

### 3.4 Acute and late toxicities of normal tissues

As for acute toxicities, grade 3 mucositis was observed in 38 patients. No grade 4+ mucositis or grade ≥2 dermatitis was observed. Late toxicities of grade ≥3 were observed in 13 patients (Table 4), including grade 3 osteoradionecrosis (ORN) in 9 of them. The distribution of grade 4 late toxicities was as follows: mucositis (n = 1), brain injury (n = 1), retinopathy (n = 1), and radiation‐induced malignancy (n = 1). A case of grade 4 mucositis occurred in a 73‐year‐old female patient who had postoperative recurrence of mucoepidermoid carcinoma of the palate (rT2N0M0). C‐ion RT was delivered at 65 Gy (RBE) in 26 fractions using four ports after chemotherapy (the type of anticancer drug is unknown). Grade 4 mucositis appeared 3.5 months after C‐ion RT. Forty‐eight months after irradiation, the tumor was controlled and no distant metastasis was observed. A case of grade 4 brain injury and retinopathy occurred in a 37‐year‐old female patient who had adenoid cystic carcinoma of the maxillary gingiva (T1N0M0). C‐ion RT was delivered at 65 Gy (RBE) in 26 fractions using 5 ports. Brain metastasis appeared 2.6 months after C‐ion RT. Grade 4 retinopathy and brain injury occurred 20 and 35 months after C‐ion RT, respectively. There was no evidence of local recurrence 53 months after C‐ion RT. A case of grade 4 radiation‐induced second malignancy occurred in a 70‐year‐old male patient who had mucosal melanoma of the oral cavity (T3N0M0) treated with C‐ion RT. C‐ion RT using 5 ports was administered to deliver 57.6 Gy (RBE) in 16 fractions over 4 weeks combined with concurrent chemotherapy based on DTIC (120 mg/m^2^, days 1‐5), nimustine hydrochloride (70 mg/m^2^, day 1) and vincristine (70 mg/m^2^, day 1). There is no evidence of locoregional recurrence and distant metastasis 68 months after C‐ion RT, however, the patient developed sarcoma as grade 4 radiation‐induced malignancy. No grade 5 late toxicity occurred.

**Table 4:** Late toxicities (grade ≥2)

Type of toxicity	Grade 2	Grade 3	Grade 4	Grade 5	Total
Mucositis	3	0	1	0	4
Osteoradionecrosis	16	9	0	0	25
Brain injury	3	0	1	0	4
Facial nerve disorder	1	0			1
Trismus	1	0			1
Edema face	1	0			1
Retinopathy	0	0	1		1
Radiation‐induced malignancy	0	0	1	0	1

## 4 DISCUSSION

The standard treatment for resectable oral nonsquamous cell carcinoma is surgical treatment, and effective therapeutic results have been reported. The standard treatment option for unresectable or inoperable oral nonsquamous cell carcinoma is photon RT,^2^, ^3^, ^12^ although nonsquamous cell carcinomas are mostly radioresistant and associated with poor outcomes.^4^, ^5^ To date, the effectiveness of photon RT for oral nonsquamous cell carcinoma has not been extensively studied. In the meantime, C‐ion RT has already been proven as a promising treatment for radioresistant tumors,^7^, ^8^, ^9^, ^10^, ^11^, ^13^, ^14^ and its therapeutic effects for oral nonsquamous cell carcinomas have been reported.^13^, ^14^ Although several studies have examined the clinical results of photon RT or C‐ion RT for oral nonsquamous cell carcinomas, most published series were designed retrospectively, consisting of small sample sizes from single institutes. To the best of our knowledge, this is the first multicenter study, and is the largest analysis of photon RT or C‐ion RT for oral nonsquamous cell carcinoma. In this multicenter study, C‐ion RT demonstrated promising outcomes with acceptable toxicities in the treatment of oral nonsquamous cell carcinomas.

Intraoral minor SGCs are predominantly treated with surgery as well as adjuvant RT if deemed necessary, and the reported 5‐year rates of local control and survival are 73.8‐83.1% and 71.8‐73%, respectively.^15^, ^16^, ^17^ However, surgical resection is often difficult for intraoral minor SGCs cases with extraoral extension (eg, into the intracranial space or masticator space). As adjuvant or definitive treatment for intraoral minor SGCs, in spite of the still limited data regarding clinical outcomes, photon RT has become a widely used potion. Yorozu et al^4^ reported that the 5‐year local control and OS rates were 54% and 63%, respectively, among 12 patients with intraoral minor SGCs treated with photon RT, although 3 had undergone the RT postoperatively. As for brachytherapy, Huang et al^18^ reported the clinical results of ^125^I brachytherapy alone in 38 patients with recurrent or locally advanced oral and maxillofacial adenoid cystic carcinoma (including 12 with adenoid cystic carcinoma of the oral cavity). The 5‐year local control and OS rates were 59% and 65%, respectively. Fast neutron therapy, which has a similar RBE to C‐ion RT,^6^ has been reported to have therapeutic effects on intraoral minor SGC, which is known to be radioresistant. Douglas et al^19^ reported a series of 151 patients with adenoid cystic carcinoma of the head and neck who were treated with fast neutron therapy. In their study, 5‐year locoregional control and OS were 68% and 83%, respectively, among 26 patients with intraoral tumors. In the present multicenter study, C‐ion RT was found to be an effective treatment for intraoral minor SGCs, presenting a result similar to fast neutron therapy, with 3‐year local control and OS of 85.0% and 90.8%, respectively. Our findings highlight the possibility of C‐ion RT being a viable treatment option for intraoral minor SGCs, and especially for inoperable tumors.

The rare disease of mucosal melanoma of the oral cavity has consistently been associated with a poor outcome. Surveillance, epidemiology, and end results data from the period of 1973‐2012 have indicated a 5‐year OS rate of approximately 25% among patients with oral mucosal melanoma.^20^ Complete surgical resection with clear margins is the pillar of oral mucosal melanoma management, and optimal results might be expected. However, it is frequently difficult for oral mucosal melanoma to keep a tumor‐free surgical margin of 1‐2 cm, which is generally required and accepted for cutaneous melanoma, as the extent of histopathologically assessed tumor expanse is usually greater than that of the gross disease, and it might include the pharynx and paranasal sinus. In the case of surgical therapy, Lopez‐Graniel et al^21^ reported a 5‐year OS rate of 6.6% for 15 oral mucosal melanoma patients, and Tanaka et al^22^ reported a 5‐year OS rate of 15.4% for 13 primary oral mucosal melanoma patients. However, for inoperable cases, Wushou et al^5^ reported a 3‐year OS rate of 0% in 21 patients treated with photon RT alone. The therapeutic effect of fast neutron therapy on primary mucosal melanomas of the head and neck of 14 patients (including 3 with oral mucosal melanomas) as reported by Liao^23^ resulted in 5‐year local control and OS rates of 66% and 21%, respectively. However, their report did not describe the outcomes of the patients in detail. In comparison, the current study showed 3‐year local control and OS of patients with oral mucosal melanoma to be 93.3% and 62.0%, respectively. Thus, C‐ion RT attained superior local control and positive survival benefits compared with the historical data of photon RT.

A retrospective multicenter study of C‐ion RT for head and neck mucosal melanoma has already shown overall survival benefit with use of DTIC‐based concurrent chemotherapy. In this study, concurrent chemotherapy was administered for 17 mucosal melanoma patients but there was no significant improvement in overall survival. It has recently been reported that ipilimumab (cytotoxic T‐lymphocyte‐associated antigen‐4 checkpoint inhibitor) and nivolumab (programmed death‐1 checkpoint inhibitor) offer complementary activities against melanoma. According to Karlsson et al checkpoint inhibitors outperform immunotherapy or chemotherapy in terms of survival and tumor response in patients with stage III/IV unresectable cutaneous melanoma.^24^ Thus C‐ion RT in combination with immunotherapy may improve treatment outcomes in patients with oral mucosal melanoma.

ORN is one of the critical complications in patients with oral malignancies who are treated with RT. Kuhnt et al^23^ reported that 13.6% of 256 patients with oral cancer developed severe ORN that required extensive surgical intervention after photon RT. Maor et al^25^ reported that, among 68 patients with advanced head and neck cancer (including 21 with oral cancer) who were treated with fast neutron therapy, ≥ grade 3 late toxicity was observed in 39.7%. In their study, grade 4 ORN occurred in 4 patients (5.9%). Liao et al^26^ reported that 14.3% of 14 patients with mucosal melanoma of the head and neck (including 3 oral mucosal melanomas) developed severe ORN requiring extensive surgical intervention after fast neutron therapy. However, in those reports of fast neutron therapy, the incidence of ORN in patients with oral malignancies was not described in detail. Chang et al^27^ reported that a radiation dose >70 Gy was associated with an increased risk for ORN. In our study, 64.0 Gy (RBE) in 16 fractions at 4 fractions per week was the most commonly prescribed dose. If the predetermined RBE value was correct and the linear‐quadratic model could be applied to C‐ion RT, 64 Gy (RBE) in 16 fractions would be equivalent to 89.6 Gy (RBE) at a fractionation of 2 Gy (RBE) per fraction with a 3‐Gy α/β value. Although this dose was definitely high compared with the standard dose for oral cancer used in photon RT, the incidence of grade 3 ORN requiring surgical intervention was 11.8%, which was similar to the reported photon RT data. C‐ion RT with its dose‐localization properties may reduce the irradiated volume of the jaw compared with photon RT.

Sasahara et al^28^ concluded, in regard to 63 head and neck tumors treated with C‐ion RT, that the risk factors for ORN of the maxilla included the presence of teeth within the PTV and a maxillary volume receiving >50Gy (RBE) in a 16‐fraction protocol. In addition, oral health conditions (periodontal pocket depth, dental plaque score, alveolar bone loss level, and radiographic periodontal status) post radiation treatment were risk factors for ORN.^29^ To reduce the risk of ORN, in case of a questionable prognosis within the PTV, total tooth extraction before starting RT was recommended.^30^ Moreover, a custom‐made mouthpiece and spacer might reduce the volume of the jawbone that would be exposed to high‐dose irradiation.^31^

In this study, T4 classification was found to be the significant risk factor for local control in multivariate analysis. Although the prescribed dose was not a significant risk factor of local control, a dose less than 64 Gy in 16 fractions (EQD2^α/β 10^ = 74.7 Gy [RBE]) was used in 4 cases of 7 cases who developed local recurrence. Of these 4 cases, 2 cases received 57.6 Gy (RBE) in 16 fractions (EQD2^α/β 10^ = 65.3Gy [RBE]), 1 case received 65 Gy (RBE) in 26 fractions (EQD2^α/β 10^ = 67.7Gy [RBE]) and 1 case received 70.4 Gy (RBE) in 26 fractions (EQD2^α/β 10^ = 74.6Gy [RBE]). Consideration of prescribed doses and fractionation could improve clinical outcome of treatment. However, increasing the prescribed dose beyond 64 Gy (RBE) in 16 fractions could be difficult in terms of safety and tolerability given the risk of osteradionecrosis and other acute and chronic toxicities.

This study has two limitations. First, it was based on retrospective data, and several doses and fractionations of C‐ion RT were included. However, no significant differences in tumor control, toxicity, or OS were noted on the basis of the dose fractionations. An integrated treatment schedule using 16 fractions over 4 weeks with a total dose of 57.6 or 64.0 Gy (RBE) has been followed in a multicenter prospective registry study of Japan since April 2016. The second limitation was the short follow‐up period (median, 31.1 months). Successive follow‐up will be necessary to confirm the long‐term efficacy and the incidence rate of late toxicity.

In conclusion, a multicenter study in Japan showed that C‐ion RT offered promising local control and outcomes in a large cohort of patients with locally advanced oral nonsquamous cell carcinomas. In the case of patients with inoperable, locally advanced oral nonsquamous cell carcinomas, C‐ion RT might be considered as a viable option. Prospective multicenter studies with longer follow‐up periods will be necessary to validate these findings.

## CONFLICT OF INTEREST

The authors declare that they have no conflicts of interests.

## Data Availability

Data openly available in a public repository that issues datasets with DOIs.

## References

[1] Daley T , Darling M . No squamous cell malignant tumours of the oral cavity: an overview. J Can Dent Assoc. 2003;69(9):577‐582. 14653933

[2] NCCN guidelines ‐ national comprehensive cancer network practice guidelines in oncology version 2.2017, pp SALI‐1 to SALI‐4, 2017. https://www.nccn.org/professionals/physician_gls/f_guidelines.asp. Accessed July 7, 2017.

[3] NCCN guidelines ‐ national comprehensive cancer network practice guidelines in oncology version 2.2017, pp MM‐1 to MM‐4, 2017. https://www.nccn.org/professionals/physician_gls/f_guidelines.asp. Accessed July 7, 2017.

[4] Yorozu A , Sykes AJ , Slevin NJ . Carcinoma of the hard palate treated with radiotherapy: a retrospective review of 31 cases. Oral Oncol. 2001;37(6):493‐497. 11435175 10.1016/s1368-8375(00)00136-6

[5] Wushou A , Zhao YJ . The management and site‐specific prognostic factors of primary oral mucosal malignant melanoma. J Craniofac Surg. 2015;26(2):430‐434. 25668115 10.1097/SCS.0000000000001328

[6] Inaniwa T , Kanematsu N , Matsufuji N , et al. Reformulation of a clinical‐dose system for carbon‐ion radiotherapy treatment planning at the National Institute of Radiological Sciences. Japan, Phys Med Biol. 2015;60(8):3271‐3286. 10.1088/0031-9155/60/8/3271 25826534

[7] Suefuji H , Koto M , Demizu Y , et al. A Retrospective Multicenter Study of Carbon Ion Radiotherapy for Locally Advanced Olfactory Neuroblastomas. Anticancer Res. 2018;38(3):1665‐1670. 29491100 10.21873/anticanres.12399

[8] Sulaiman NS , Demizu Y , Koto M , et al. Multicenter Study of Carbon‐Ion Radiation Therapy for Adenoid Cystic Carcinoma of the Head and Neck: Subanalysis of the Japan Carbon‐Ion Radiation Oncology Study Group (J‐CROS) Study (1402 HN). Int J Radiat Oncol Biol Phys. 2018;100(3):639‐646. 29413278 10.1016/j.ijrobp.2017.11.010

[9] Saitoh J‐I , Koto M , Demizu Y , et al. A Multicenter Study of Carbon‐Ion Radiation Therapy for Head and Neck Adenocarcinoma. Int J Radiat Oncol Biol Phys. 2017;99(2):442‐449. 28871995 10.1016/j.ijrobp.2017.04.032

[10] Shirai K , Koto M , Demizu Y , et al. Multi‐institutional retrospective study of mucoepidermoid carcinoma treated with carbon‐ion radiotherapy. Cancer Sci. 2017;108(7):1447‐1451. 28474791 10.1111/cas.13270 PMC5497800

[11] Koto M , Demizu Y , Saitoh J‐I , et al. Multicenter Study of Carbon‐Ion Radiation Therapy for Mucosal Melanoma of the Head and Neck: Subanalysis of the Japan Carbon‐Ion Radiation Oncology Study Group (J‐CROS) Study (1402 HN). Int J Radiat Oncol Biol Phys. 2017;97(5):1054‐1060. 28332989 10.1016/j.ijrobp.2016.12.028

[12] NCCNguidelines – national comprehensive cancer network practice guidelines in oncology version 2.2017, pp MS‐18, 2017. https://www.nccn.org/professionals/physician_gls/f_guidelines.asp. Accessed July 7, 2017.

[13] Naganawa K , Koto M , Takagi R , et al. Long‐term outcomes after carbon‐ion radiotherapy for oral mucosal malignant melanoma. J Radiat Res. 2017;58(4):517‐522. 28028129 10.1093/jrr/rrw117 PMC5570020

[14] Ikawa H , Koto M , Hayashi K , et al. Feasibility of carbon‐ion radiotherapy for oral non‐squamous cell carcinomas. Head Neck. 2019;41(6):1795‐1803. 30676669 10.1002/hed.25618 PMC6590439

[15] Copelli C , Bianchi B , Ferrari S , Ferri A , Sesenna E . Malignant tumors of intraoral minor salivary glands. Oral Oncol. 2008;44(7):658‐663. 17996484 10.1016/j.oraloncology.2007.08.018

[16] Mücke T , Robitzky LK , Kesting MR , et al. Advanced malignant minor salivary glands tumors of the oral cavity. Oral Surg Oral Med Oral Pathol Oral Radiol Endod. 2009;108(1):81‐89. 19386516 10.1016/j.tripleo.2009.01.013

[17] Agarwal JP , Jain S , Gupta T , et al. Intraoral adenoid cystic carcinoma: prognostic factors and outcome. Oral Oncol. 2008;44(10):986‐993. 18329324 10.1016/j.oraloncology.2008.01.004

[18] Huang M‐W , Zheng L , Liu S‐M , et al. 125I brachytherapy alone for recurrent or locally advanced adenoid cystic carcinoma of the oral and maxillofacial region. Strahlenther Onkol. 2013;189(6):502‐507. 23625361 10.1007/s00066-013-0324-3

[19] Douglas JG , Laramore GE , Austin‐Seymour M , Koh W , Stelzer K , Griffin TW . Treatment of locally advanced adenoid cystic carcinoma of the head and neck with neutron radiotherapy. Int J Radiat Oncol Biol Phys. 2000;46(3):551‐557. 10701733 10.1016/s0360-3016(99)00445-9

[20] Lee RJ , Lee SA , Lin T , Lee KK , Christensen RE . Determining the epidemiologic, outcome, and prognostic factors of oral malignant melanoma by using the Surveillance, Epidemiology, and End Results database. J Am Dent Assoc. 2017;148(5):288‐297. 28325493 10.1016/j.adaj.2017.01.019

[21] Lopez‐Graniel CM , Ochoa‐Carrillo FJ , Meneses‐García A . Malignant melanoma of the oral cavity: diagnosis and treatment experience in a Mexican population. Oral Oncol. 1999;35(4):425‐430. 10645410 10.1016/s1368-8375(99)00017-2

[22] Tanaka N , Mimura M , Ogi K , Amagasa T . Primary malignant melanoma of the oral cavity: assessment of outcome from the clinical records of 35 patients. Int J Oral Maxillofac Surg. 2004;33(8):761‐765. 15556323 10.1016/j.ijom.2004.01.008

[23] Liao JJ , Parvathaneni U , Laramore GE , et al. Fast neutron radiotherapy for primary mucosal melanomas of the head and neck. Head Neck. 2014;36(8):1162‐1167. 23852725 10.1002/hed.23428

[24] Karlsson AK , Saleh SN . Checkpoint inhibitors for malignant melanoma: a systematic review and meta‐analysis. Clin Cosmet Investig Dermatol. 2017;10:325‐339. 10.2147/CCID.S120877 PMC5580705 28883738

[25] Maor MH , Errington RDouglas , Caplan RJ , et al. Fast‐neutron therapy in advanced head and neck cancer: a collaborative international randomized trial. Int J Radiat Oncol Biol Phys. 1995;32(3):599‐604. 7790244 10.1016/0360-3016(94)00595-C

[26] Kuhnt T , Stang A , Wienke A , Vordermark D , Schweyen R , Hey J . Potential risk factors for jaw osteoradionecrosis after radiotherapy for head and neck cancer. Radiat Oncol. 2016;11:101. 27473433 10.1186/s13014-016-0679-6 PMC4967325

[27] Chang DT , Sandow PR , Morris CG , et al. Do pre‐irradiation dental extractions reduce the risk of osteoradionecrosis of the mandible? Head Neck. 2007;29(6):528‐536. 17230555 10.1002/hed.20538

[28] Sasahara GO , Koto M , Ikawa H , et al. Effects of the dose‐volume relationship on and risk factors for maxillary osteoradionecrosis after carbon ion radiotherapy. Radiat Oncol. 2014;9(1):92. 24708583 10.1186/1748-717X-9-92 PMC3992144

[29] Katsura K , Sasai K , Sato K , Saito M , Hoshina H , Hayashi T . Relationship between oral health status and development of osteoradionecrosis of the mandible: a retrospective longitudinal study. Oral Surg Oral Med Oral Pathol Oral Radiol Endod. 2008;105(6):731‐738. 18329913 10.1016/j.tripleo.2007.10.011

[30] Schiødt M , Hermund NU . Management of oral disease prior to radiation therapy. Support Care Cancer. 2002;10(1):40‐43. 11777187 10.1007/s005200100284

[31] Ikawa H , Koto M , Ebner DK , et al. The efficacy of custom‐made mouthpiece with spacer to reduce osteoradionecrosis in carbon‐ion radiotherapy for tongue‐base tumor. Adv Radiat Oncol. 2019;4:15‐19. 30706004 10.1016/j.adro.2018.08.016 PMC6349587

